# Molecular modeling study on the water-electrode surface interaction in hydrovoltaic energy

**DOI:** 10.1038/s41598-023-39888-8

**Published:** 2023-08-07

**Authors:** Goncagül Serdaroğlu, İ. Afşin Kariper, S. Esra Bolsu Kariper

**Affiliations:** 1 Mathematics and Science Education, Faculty of Education, Sivas Cumhuriyet University, Sivas, Turkey; 2 Education Faculty, Erciyes University, Kayseri, Turkey; 3 Erciyes Teknopark, Building 1, No. 41, Kayseri, Turkey; 4 Vocational School of Health Services, Avrasya University, Trabzon, Turkey

**Keywords:** Chemistry, Energy science and technology

## Abstract

The global energy problem caused by the decrease in fossil fuel sources, which have negative effects on human health and the environment, has made it necessary to research alternative energy sources. Renewable energy sources are more advantageous than fossil fuels because they are unlimited in quantity, do not cause great harm to the environment, are safe, and create economic value by reducing foreign dependency because they are obtained from natural resources. With nanotechnology, which enables the development of different technologies to meet energy needs, low-cost and environmentally friendly systems with high energy conversion efficiency are developed. Renewable energy production studies have focused on the development of hydrovoltaic technologies, in which electrical energy is produced by making use of the evaporation of natural water, which is the most abundant in the world. By using nanomaterials such as graphene, carbon nanoparticles, carbon nanotubes, and conductive polymers, hydrovoltaic technology provides systems with high energy conversion performance and low cost, which can directly convert the thermal energy resulting from the evaporation of water into electrical energy. The effect of the presence of water on the generation of energy via the interactions between the ion(s) and the liquid–solid surface can be enlightened by the mechanism of the hydovoltaic effect. Here, we simply try to get some tricky information underlying the hydrovoltaic effect by using DFT/B3LYP/6-311G(d, p) computations. Namely, the physicochemical and electronic properties of the graphene surface with a water molecule were investigated, and how/how much these quantities (or parameters) changed in case of the water molecule contained an equal number of charges were analyzed. In these computations, an excess of both positive charge and negative charge, and also a neutral environment was considered by using the Na^+^, Cl^−^, and NaCl salt, respectively.

## Introduction

Natural water is the most abundant clean and sustainable resource on earth, covering approximately 71% of the earth's surface and capturing approximately 70% of the solar energy received by the earth's surface^1^. The excess energy in the water, which can flow and evaporate spontaneously by adsorbing thermal energy without being affected by environmental conditions, is separated from the surface in the form of latent heat^2,3^. The evaporation potential and current, which continues as long as the evaporation of water continues, provide the conversion of thermal energy into electrical energy^2,4^. Due to the unique characteristics of both the water molecule and graphene surface in terms of the structural, electronic, and thermal properties, water-graphene systems have been increasingly studied in many areas^5–7^. The graphene structure consists of the *sp*^*2*^ hybridized carbon atoms and thus the electron delocalization on the surface plane is responsible for the in-plane heat conductivity, while out-of-plane thermal conductivity can be provided by the weak dispersion forces or van der Waals interactions^8^. Also, the water molecule has been reported with a vital role in the harvesting of new-generation energy such as kinetic energy by hydrovoltaic tools, gradient, and evaporation energy^9^.

Hydrovoltaic systems, which produce electricity from the direct interaction of water with the material, are preferred instead of existing technologies used to obtain electrical energy from the kinetic energy of water. Energy from water is converted into electrical energy by hydrovoltaic effects provided by mechanisms such as flow potential, fluctuation potential, shrinkage potential and evaporation-induced electricity, and gradient-induced ion diffusion. When solids come into contact with water, ions with opposite charges to the surface charge are attracted and an electrical double layer (EDL) is formed at the water–solid interface. EDL-based electrokinetic effects, such as flow potential, can convert the kinetic energy of water flowing through narrow channels into electrical energy. However, the formation of a high flow potential is possible with a large pressure gradient. By placing a single layer of graphene or carbon on the film formed along the surface of the rippling water, wave energy is collected and the wave potential is converted into electrical energy. A drop of water is dripped onto a graphene sheet to create an attractive potential and is used to collect the kinetic energy of raindrops and solar cells operating on rainy days. Evaporation of water in our environment always takes place as a normal process, during which a large amount of thermal energy is converted into latent water vapor energy. Sustainable tension can be obtained from the evaporation of natural water using a small porous piece of carbon black film partially immersed in water. Any energy gradient is equivalent to a repulsive force that can do work on the system, such as moving charge carriers. Osmotic energy is the most common gradient energy and is derived from the directional diffusion of ions along a concentration gradient in water. Low ion concentration and the large distance between ions and solid surface limit the interaction between water vapor and solid surface, resulting in low electrical output^9^. Electric output generation can be improved using porous materials such as carbon black sheets and nanowires with nano-sized channels^10,11^. In this direction, studies have focused on finding the amount of energy the system has due to the effect on the water drop and graphene surface.

Recently, molecular dynamic computations were performed to explore the effect of the water humidity on the graphitic surface and suggested that the graphene surface is quite wettable and presents hydrophobic behavior in case it was covered by the double water layers^12^. The hydrophilic and hydrophobic phenomena have great importance in predicting/evaluating the interactions that exist in a specific adsorbent (a solid surface)-adsorbed (water)^13–15^. In this respect, Jiang et al. explored the catalytic effect of a perpendicular electric field on the reversible transition of graphene with water from hydrophobic to hydrophilic by using first-principles calculations; they disclosed that the electric field could act as a switch to reversibly change the graphene from hydrophobic to hydrophilic in the presence of water vapor^16^. On the other hand, polarizability holds a crucial position in the modulation of its hydrophilicity-hydrophobicity dynamics, establishing the groundwork for its interactional paradigm with water molecules. Thus, this intrinsic characteristic significantly influences graphene's physical properties, thus broadening the potential for its applicability across a variety of scientific domains. In this regard, a comprehensive analysis of graphene's electronic polarizability had been conducted using multiple theoretical methodologies, including Kubo's linear response formalism for frequency and wave-vector-dependent polarizability within the random phase approximation^17^. Furthermore, the first-principles density functional approach was used to analyze the properties of graphene multilayers and graphite under an applied electric field; the specific regions of the designed graphene multilayers were explored in terms of the induced charge under applied electric fields^18^. In a study, an analytical model, in which parameters such as polarizability and bending stiffness were derived from numerical fits to simulation data was introduced to predict the alignment of infinitely wide Graphene Nanoribbons (GNRs) to an electrostatic field; the model revealed that there was a linear correlation between alignment angle, field strength square, and graphene length for small deformations^19^. Recently, Escalona et al.^20^ examined the water-graphene interface in distinct systems, one with graphene in water and another with a water droplet on graphene, under an electric field; their MD results implied that water's orientation near graphene was affected by graphene's polarizability, particularly under an electric field.

In the case of the adsorption of the water molecule issue has a complexity because of the possible H-bonding between the surface and water molecule or between the water molecules. Hence, it is hard to evaluate this kind of interaction just only using a limited factor such as H-bonding tendency, surface properties, hydrophilicity, etc.^21^. In the past, Monte Carlo simulations were conducted to elucidate the water adsorption on the graphene surface at various temperatures at 313, 316, and 323 K, and the results showed that water molecules would prefer to merge at the edges of the graphene surface rather than adsorb onto the basal graphene plane^22^. Moreover, an ab initio study is reported to investigate how many electrons each ion can attract from the polymer (PET)-enclosed graphene surface and whether it is suitable for harvesting water energy^23^. Accordingly, the authors suggested that the shortened surface thickness or more substrate dipole can boost the output power^23^. In a remarkable, Yin et all demonstrated that the electrokinetic potential can be produced in a graphene sheet that moves between air and solution interfaces, and the results of that work have quite promised to design self-powered sensors and monitors^24^. In designing smart materials, especially, graphene-based systems are getting great attention because of promising structural relevant properties such as mechanical and thermal features, and applicable surface area^25^.

One of the main issues in the contemporary age, even the most important, is to generate energy from ergonomic, economical, renewable, etc., then for a green future. Nowadays, waste energy is also processed to re-generate energy, but research has shifted towards nanogenerators due to these (secondary) energies obtained being very low efficiency. Although the three main mechanisms of the hydovoltaic nanogenerators, as a class of nanogenerators, have been known, the water effect on the solid surface (electrode) has not been explained and keep stand as controversial. In this respect, the reported data in the literature are generally related to developing new electrodes that can harvest hydrovoltaic energy, but not to deeper reasons for them, especially electronic structures and relevant electronic properties.

The finite-dimensional constraints of graphene have noteworthy implications on the manifestation of the hydrovoltaic effect, an outcome that diverges markedly when considered in the context of a hypothetically infinitely extended graphene plane. The existence of boundary effects and the limited expanse in these finite graphene constructs may notably modulate the interactions and spatial distribution of ions and water molecules on the graphene interface, leading to consequential influences on hydrovoltaic energy generation. As reported, the simulation outcomes demonstrate that water exhibits a preference for adsorption around the functional groups of graphene, fostering the formation of clusters^17^. These clusters subsequently expand and coalesce at the peripheries of the graphene layers, instead of adsorbing onto the graphene's basal planes. This phenomenon can be attributed to the stronger electrostatic interactions, particularly hydrogen bonding, between water molecules, which supersede the relatively weaker dispersion interactions with the basal planes of graphene^17^.

While the theoretical exploration of infinite graphene planes presents an intellectually engaging discourse, it is essential to acknowledge that the preponderance of graphene's practical implementations is based on finite-sized components. Consequently, despite the intrinsic limitations inherent to the finite nature of these graphene-based systems, the insights garnered from such studies continue to offer substantial value and applicability across multiple domains.

Until now, DFT computations in terms of molecular mechanics and dynamic perspective have been applied successfully to graphene/ graphite systems^26–30^ such as doped by metal or transition metal, nanostructures, or graphene quantum dots, etc. For instance, the Density Functional Theory (DFT) based on first principles examined the impact of different impurities on the structure, electronics, and charge transfer in graphene to explore the possible usage for graphene in optoelectronics and other devices requiring switching capabilities^31^. Other first-principles DFT computations were reported to explore the gas-sensing performance of transition metal-doped graphene systems^32^. Furthermore, Kumar et al. employed DFT computations to examine the impact of doping graphene with platinum group elements (PGE); they calculated the energy gap, UV–vis absorption spectra, and Mulliken population to explore the potential uses in wearable electronics, optoelectronics, sensors, and solar cells^33^. Furthermore, the graphene oxide nanosheet (GON) was modified based on the Lerf-Klinowski model and investigated the impact of integrating Be, B, N, O, and F atoms on GON's properties by using the B3LYP-D3/6–31 + G(d,p)^34^. Also, the DFT calculations and MD simulations were utilized to investigate boron-modified graphane nanoparticles to determine the reactivity and hydrogen binding potencies to examine their optoelectronic properties, indicating potential applications in clean energy and materials science^35^. In another study on research of BX (X = N, P) doped twin-graphene, the authors utilized the DFT based on first-principle calculations to explore the possible potentials of the designed systems, which would produce clean energy^36^.

To understand the basic mechanism of the surface-based hydrovoltaic effect; it is necessary to understand the interactions and energy relations of water, ions, and electrons on the solid–liquid surface. In this direction, in our study, the physicochemical effects of a water molecule on the graphene surface as a typical model were investigated. In addition, it also analyzed how the physicochemical parameters change when the water molecule contains an equal number of charges, excess positive charge or excess negative charge. Our work serves as a significant step toward understanding the intricate dynamics at the solid–liquid interface, crucial for optimizing the hydrovoltaic effect in various applications. By providing detailed insights into the physicochemical interactions between the ions and water and finite graphene, we hope that the results of this work pave the way for future research aimed at enhancing the efficiency and performance of graphene-based hydrovoltaic devices.

## DFT calculations

All electronic structure computations were performed by G09^37^ package and analyses and representations of the results were made by using GaussView 6.0.16^38^. At first, the electronic structures of isolated molecules, ions, and salt were confirmed by the absence of no imaginary frequency following the geometry optimization by the default convergence criteria^39,40^, at B3LYP/6-311G** level^41,42^ and basis set^43–45^.

The thermochemical and physical characteristics of all studied species were determined by the quantum statistical principles^46–48^. As known well, thermodynamic and thermochemical properties for the relevant systems are calculated by using the total partition function which depends on the freedom degrees of the translational (Q_trans._), rotational (Q_rot._), vibrational (Q_vib._), and electronic (Q_elec._) movements^46,47^. For a general system, the molecular partition function is defined as

1 $$Q = Q_{trans.} \times Q_{rot.} \times Q_{vib. } Q_{elec.}$$

The vibrational partition function for the asymmetric top molecules is given below, and the degrees of vibrational freedom contribution to the thermochemical quantities can be calculated by Eqs. (3)–(5)^46–49^.

2 $$Q_{vib.} = \mathop \prod \limits_{j = 1}^{3N - 6} \frac{{e^{{ - \Theta_{v,j} /2T}} }}{{\left( {1 - e^{{ - \frac{\Theta v,j}{T}}} } \right)}}$$

3 $$E_{vib.} = Nk \mathop \sum \limits_{j = 1}^{3N - 6} \left( {\frac{{\Theta_{v,j} }}{2} + \frac{{\Theta_{vj} e^{{ - \Theta_{v,j} /T}} }}{{\left( {1 - e^{{ - \frac{\Theta v,j}{T}}} } \right)}}} \right)$$

4 $$S_{vib} = Nk\mathop \sum \limits_{j = 1}^{3N - 6} \left[ {\frac{{\Theta_{v,j} /T}}{{\left( {e^{{\Theta_{v,j} /T}} - 1} \right)}} - \ln \left( {1 - e^{{ - \Theta_{v,j} /T}} } \right)} \right]$$

5 $$Cv_{vib.} = Nk \mathop \sum \limits_{j = 1}^{3N - 6} \left[ {\left( {\frac{{\Theta_{vj} }}{T}} \right)^{2} \frac{{e^{{\Theta_{vj/T} }} }}{{\left( {e^{{\Theta_{v,j} /T}} - 1} \right)^{2} }}} \right]$$

Here*, E*_*vib*_. “vibrational thermal energy”, *S*_*vib*_*.* “vibrational entropy”, and *Cv*_*vib*_. “vibrational heat capacity” is defined as^25–29^. The terms are expressed as $\Theta_{vj} = \frac{{hv_{j} }}{k}$ “the vibrational temperature”, h → “Planck constant”, k → “Boltzmann constant”, and *ν*_*j*_ → ” *j*th fundamental frequency”.

Furthermore, the values *I* “ionization energy” and *A* “electron affinity”^50^ are used for determining the reactivity properties by using the following equations.

$$\begin{aligned} I & = - {\text{E}}_{{{\text{HOMO}}}} \\ A & = - {\text{E}}_{{{\text{LUMO}}}} \\ \chi & = - \left( {\frac{I + A}{2}} \right) \\ \eta & = \frac{I - A}{2} \\ \omega & = \frac{{\mu^{2} }}{{2{\upeta }}} \\ \Delta N_{max} & = \frac{I + A}{{2\left( {I - A} \right)}} \\ \omega^{ + } & \approx (I + 3A)^{2} /\left( {16\left( {I - A} \right)} \right) \\ \omega^{ - } & \approx (3I + A)^{2} /\left( {16\left( {I - A} \right)} \right) \\ \Delta \varepsilon_{back - donation} & = - \frac{\eta }{4} \\ \end{aligned}$$

Here, χ → “electronic chemical potential” η → “global hardness”, ω → “electrophilicity”, ΔN_max_ → “the maximum charge transfers capability index”^51–56^, ω- “the electrodonating power” and ω + “the electroaccepting power”^57^, and ΔE_back-donat._ “back-donation energy”^58^.

## Result and discussion

### Molecule geometry and physicochemical properties

**Figure 1:**
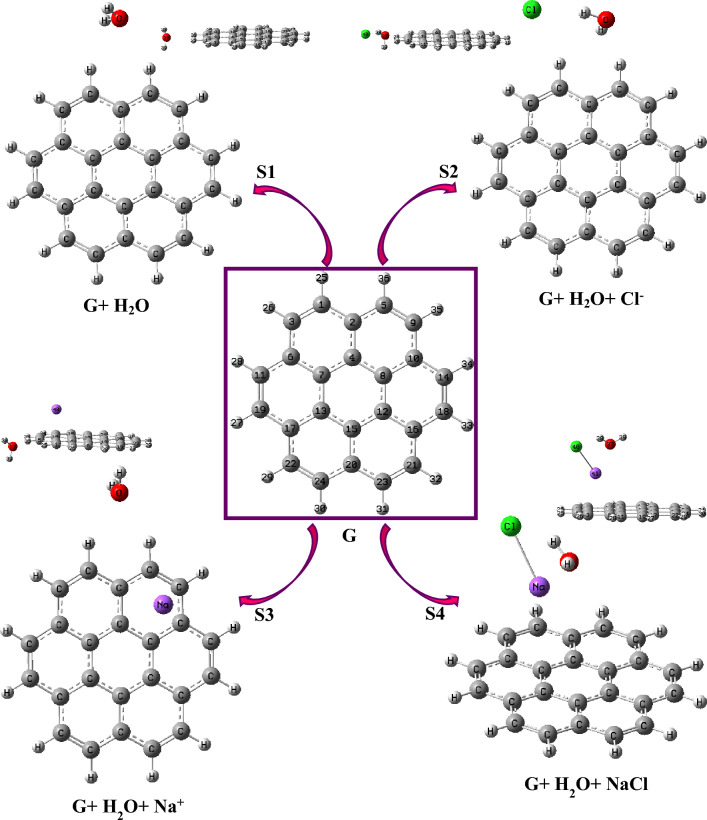
The optimized systems of the possible interaction(s) of Graphene. The atom numbering scheme of the four systems is the same as those of the Graphene (**G**) core structure (The optimized structures were visualized by using the GaussView 6.0.16^38^ software, took a screenshot, and arranged by the word (MS) tools).

The confirmed structures of the possible interactions between Graphene and anionic, cationic, salt, and water were presented in Fig. 1. Also, the calculated geometric data for each system designed were summarized in Table 1.

**Table 1:** The selected optimized parameters for the systems S1–S4 and G at B3LYP/6-311G(d,p) basis set.

	G	S1	S2	S3	S4
Bond length (Å)					
C1–C2	1.42	1.42	1.42	1.43	1.43
C2–C5	1.42	1.42	1.43	1.43	1.43
Non-bonding length (Å)					
*O*–*H*–C1	–	2.50	2.23	2.29	5.57
*Na*^+^–C5	–	–	–	2.70	3.92
*Cl*^–^–*H*–C1	–	–	2.61	–	4.78
Na^+^–C3	–	–	–	4.38	2.89
*Cl*^–^–C3	–	–	4.10	–	4.58
*Na*^+^–O	–	–	–	5.23	2.27
*O*–C4	–	–	–	–	4.49
*Bond angle (°)*					
C1–C2–C4	118.8	118.8	119.3	119.0	118.7
C1–C2–C5	122.5	122.1	121.7	122.3	122.5
C2–C4–C7	120.0	120.0	120.0	120.0	120.1
Non-bonding angle (°)					
C3–C1–*Cl*^*−*^	–	–	100.8	–	77.2
C2–C5–*O*	–	99.5	108.2	98.3	61.7
C1–C2–*Na*^+^	–	–	–	120.9	70.9
C4–C2–*O*	–	179.0	169.8	176.3	72.1
C8–C4–*Na*	–	–	–	75.7	122.3
C4–*Na*–Cl	–	–	–	–	166.5
Dihedral angle (°)					
C1–C2–C4–C7	−0.0	−0.0	0.0	0.1	−0.1
C3–C1–C2–C5	180.0	180.0	−179.8	179.5	−179.6
C2–C5–H–*O*	–	0.7	−28.5	−6.7	69.6
C2–C1−H–*Cl*^*−*^	–	–	−171.1	–	−105.5
H–C1–C2–*Na*^+^	–	–	–	90.9	112.3
C8–C4–*Na*–Cl	–	–	–	–	126.4
C5–C2–C4–*O*	–	–	–	–	95.1

From Table 1, all C–C lengths for systems **G** (isolated graphene) and **S1** (with water on the Graphene surface) were calculated as 1.42 Å whereas this bond for **S3** and **S4** was determined as 1.43 Å. In the past, the C–C length for the graphene unit was determined in the range of 1.34–1.49 Å^26,28,31,59–62^ depending on the type of the doped atom. Namely, the C–C bond length for the Mg-doped graphene core was calculated as 1.34–1.44 Å ^31^, whereas for the O-doped Graphene, the nearest C–C length was predicted as 1.49 Å ^33^. On the other hand, this length for the isolated graphene core was mostly determined as 1.42–1.43 Å ^59^. Also, the neighbor C–C bond lengths for the B, N, O, and F-doped graphene structures were estimated at 1.403, 1.416, 1.485, and 1.353 A, respectively^63^. Also, the O–H–C1 length for all systems was calculated as 2.50, 2.23, 2.29, and 5.57 Å: the oxygen distance to the graphene surface did grow longer with the presence of the salt (NaCl) while it did narrow with the existence of the ions (Na^+^ or Cl^−^). The geometric parameters show that the electronic attraction force between the Na^+^ ion and the graphene surface is larger than the other systems, and therefore the Na^+^ ion (**S3**) is placed parallel to the graphene surface. On the other hand, the distance between the Cl^-^ ion and the graphene surface increased due to the electronic repulsion forces between the charge density on the graphene surface and the charge cloud of the Cl^-^ ion, and *Cl*^–^–* H*-C1 length was calculated as 2.61 Å. Also**,** Na^+^–C3 and *Cl*^–^–C3 lengths were calculated at 4.38 and 4.10 Å, respectively, whereas the Na^+^–C2 and *Cl*^–^–C2 distances were calculated at 2.71 and 4.78 Å. The C1-C2 –Na + and C8–C4–Na angles for **S3**, which the placement of the Na + ion on the graphene-like layer, were calculated as 120.9° and 75.7°, respectively. Moreover, the C4–C2–O angle for **S1** was calculated as 179.0° which implied that the water molecule was positioned approximately at the same plane with the graphene structure, whereas this angle for **S4** was calculated as 72.1° which was a sign that the water molecule (existence of the salt) was placed on the surface at an angle. In addition, the C2–C5–O angle for systems S1–S4 was calculated at 99.5°, 108.2°, 98.3°, and 61.7°, respectively. From Table 1, all C–C–C angles for the single-layer graphene unit were calculated in the range of 118.8–122.5°, as can be expected from *sp*^*2*^ hybridization.

Also, the calculated physical and thermochemical properties were summarized in Table 2. Accordingly, the dipole moment and polarizability values of systems **S2** and **S4** were calculated higher than those of the other systems because of the existence of the electronegative chlorine atom. Namely, the order of the dipole moments of **G** and **S1-S4** (in D unit) was determined as 22.172 (**S2**) > 10.172 (S**4**) > 9.727 (**S3**) > 2.358 (**S1**) > 0.000 **(G)**, whereas the polarizability values of them (in au) were determined as 308.940 (**S4**) > 308.878 (**S2**) > 291.786 (**S3**) > 288.305 (**S1**) > 279.745 (**G**). In the context of molecular electronic properties, our findings underscore a notable variability among the studied systems. Specifically, state S2 demonstrates the most pronounced dipole moment, standing at 22.172 D. This places S2 as the most polar entity within the studied set, implying a substantial charge separation within the molecule^64^. Contrastingly, state S4 exhibits the highest polarizability, with a measure of 308.940 atomic units. This suggests a heightened response of the electron cloud to perturbations in the external electric field, indicative of a more deformable electronic distribution under the influence of an external field^65–67^. Remarkably, ground state G is characterized by the least values for both dipole moment and polarizability, revealing its lower polarity and relative electron cloud rigidness. This set of observations facilitates a deeper understanding of the electronic characteristics of the various states, potentially driving further investigations into their interactive behaviors and reactivity profiles.

**Table 2:** The thermodynamic and physical values of systems **S1–S4, G**, and fragments at B3LYP/6-311G(d,p) basis set.

Systems	G	S1	S2	S3	S4
ΔE (au)	−921.814572	−998.245792	−1458.584231	−1160.389340	−1620.885740
ΔH (au)	−921.799460	−998.226406	−1458.563551	−1160.367981	−1620.862908
ΔG (au)	−921.854403	−998.292522	−1458.634038	−1160.437959	−1620.941907
E_therm_ (kcal/mol)	183.657	200.998	202.594	202.809	204.610
E_vib._ (kcal/mol)	181.880	199.220	200.817	201.031	202.833
Cv (cal/mol K)	64.619	75.865	80.106	81.914	85.957
Cv_vib._ (cal/mol K)	58.658	69.903	74.144	75.952	79.996
S (cal/mol K)	115.638	139.153	148.353	147.280	166.266
S_tr._ (cal/mol K)	42.994	43.167	43.478	43.375	43.666
S_rot._ (cal/mol K)	34.108	34.764	35.645	34.938	35.525
S_vib._ (cal/mol K)	38.537	61.221	69.230	68.966	87.074
μ (D)	0.000	2.358	22.172	9.727	10.172
α (au)	279.745	288.305	308.878	291.786	308.940

Fragments	H_2_O	Cl^−^	Na^+^	NaCl	
ΔE (au)	−76.426124	−460.300710	−162.081230	−622.599428	
ΔH (au)	−76.422344	−460.298350	−162.078870	−622.595766	
ΔG (au)	−76.443770	−460.315733	−162.095659	−622.621851	
E_therm._ (kcal/mol)	15.161	0.889	0.889	2.210	
E_vib._ (kcal/mol)	13.383	0.000	0.000	0.729	
Cv (cal/mol K)	6.008	2.981	2.981	6.537	
Cv_vib._ (cal/mol K)	0.046	0.000	0.000	1.569	
S (cal/mol K)	45.094	36.586	35.336	54.900	
S_tr._ (cal/mol K)	34.608	36.586	35.336	38.092	
S_rot._ (cal/mol K)	10.480	0.000	0.000	15.655	
S_vib._ (cal/mol K)	0.007	0.000	0.000	1.153	
μ (D)	2.070	0.000	0.000	9.100	
α (au)	5.915	5.121	0.445	25.638	

Thermal energy values (in kcal/mol) changed in the order of 204.610 (**S4**) > 202.809 (**S3**) > 202.594 (**S2**) > 200.998 (**S1**) > 188.657 (**G**); highly portion of it came from the vibrational motions. Also, heat capacity and vibrational heat capacity (cal/mol K) orders were calculated as of 85.957 (**S4**) > 81.914 (**S3**) > 80.106 (**S2**) > 75.865 (**S1**) > 64.619 (**G**) and 79.996 (**S4**) > 75.952 (**S3**) > 74.144 (**S2**) > 69.903 (**S1**) > 58.658 (**G**), respectively. Furthermore, the translational entropy values of the designed adsorbent-absorbance systems (S1-S4) were calculated at 43.167 (**S1**), 43.478 (**S2**), 43.375 (**S3**), and 43.666 cal/mol K (**S4**), whereas the rotational contribution to the entropy was determined at 34.764 (**S1**), 35.645 (**S2**), 34.938 (**S3**), and 35.525 cal/mol K (**S4**). As can be expected, the greatest contribution to the entropy was sourced from the vibrational freedom degree of all systems and calculated as 38.537 (**G**), 61.221 (**S**1), 69.230 (**S2**), 68.966 (**S3**), and 87.074 cal/mol K (**S4**).

The adsorption energies of the designed systems were calculated by using the following equations.

$$\begin{aligned} \Delta E_{{{\text{ads}}}} & = \Delta E_{{{\text{SYS}}}} - (\Delta E_{{\text{G}}} + \Delta E_{{\text{F}}} ) \\ \Delta H_{{{\text{ads}}}} & = \Delta H_{{{\text{SYS}}}} - (\Delta H_{{\text{G}}} + \Delta H_{{\text{F}}} ) \\ \Delta G_{{{\text{ads}}}} & = \Delta G_{{{\text{SYS}}}} - (\Delta G_{{\text{G}}} + \Delta G_{{\text{F}}} ) \\ \end{aligned}$$

where *ΔE*_SYS_ is the total energy of the defined systems; *ΔE*_G_ is the energy of the isolated graphene; *ΔE*_F_ is the sum of the energies of each isolated fragment. Similarly, *ΔH*_ads_. and *ΔG*_ads_. are defined similarly. Here, the negative energy value implied the stable adsorbate/graphene system. By using the thermochemical data given in Table 2, the calculated energies of assumed systems (**S1–S4**) were presented in Table 3. It should be expressed that each system and isolated adsorbates were optimized and confirmed to predict the thermochemical data. It is clear from Table 3 that the adsorption processes are exothermic for all systems designed here, but the energy released in the **S3** process is greater than in the others. Namely, the enthalpy change (in kJ/mol) for the assumed systems was calculated as **S1** (−12.08) < **S2** (−113.94) < **S4** (−119.03) < **S3** (−176.71); the presence of the cation Na^+^ made the enthalpy change of lowered. Furthermore, the adsorption-free energy changes indicated that the adsorption processes would occur spontaneously for the designed systems **S2–S4**. From Table 3, the adsorption-free energies of systems **S2–S4** were determined as **S2** (−52.86) < **S4** (−57.45) < **S3** (−115.86), while the free energy changing for **S1** was determined as 14.84 kJ/mol, which indicated the adsorption system could not occur spontaneously.

**Table 3:** The calculated adsorption energies (in kJ/ mol) of the designed systems at B3LYP/6-311G(d,p) basis set.

	S1	S2	S3	S4
*ΔE*	−13.38	−112.44	−177.00	−119.76
*ΔH*	−12.08	−113.94	−176.71	−119.03
*ΔG*	14.84	−52.86	−115.86	−57.45

### FMO (frontier molecular orbital) analysis

**Figure 2:**
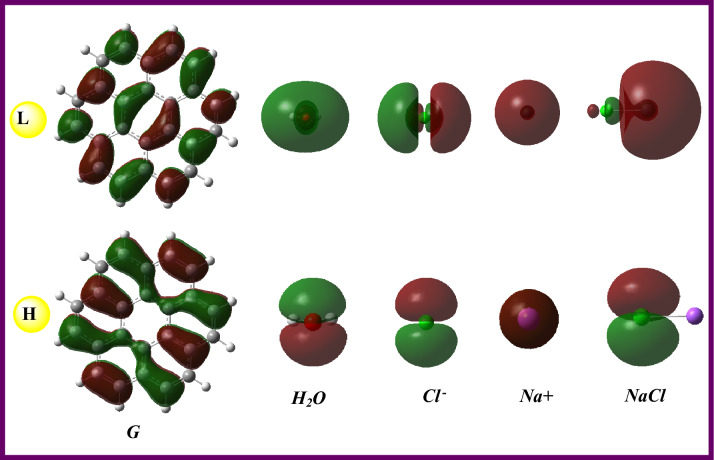
HOMO & LUMO (isoval:0.02) plots of the main components of the designed systems **S1–S4** at B3LYP/6-311G** level in gas (The HOMO & LUMO densities were analyzed and then visualized by using the GaussView 6.0.16^38^ software, took a screenshot, and arranged by the Word (MS) tools).

**Figure 3:**
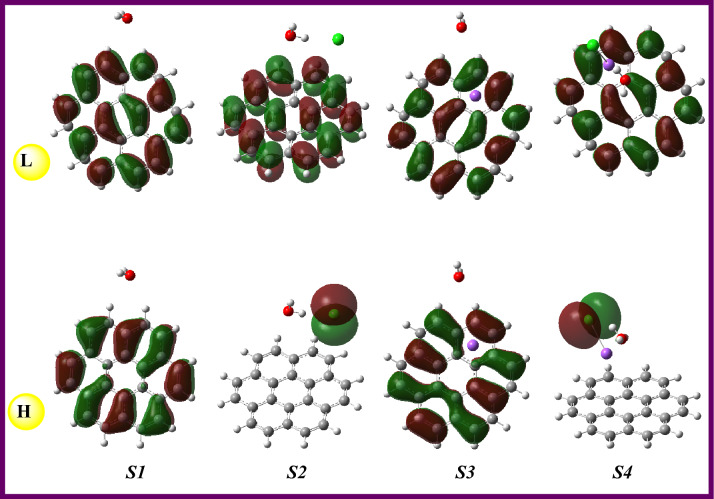
HOMO & LUMO (isoval:0.02) plots of the designed systems **S1–S4** at B3LYP/6-311G** level in gas (The HOMO & LUMO densities were analyzed and then visualized by using the GaussView 6.0.16^38^ software, took a screenshot, and arranged by the Word (MS) tool).

The possible reactivity identifiers of the isolated molecules and designed systems **S1–S4** were given in Table 4. Here, the chlorinated graphene surface can be said that the less likely to interact with an external system due to the smallest energy gap value (1.592 eV), and vice versa for the isolated graphene (4.018 eV). Considering the effect of the anion, cation, or salt on the graphene reactivity, the Na^+^ cation can be said that makes the graphene more reactive to the outer system(s) with the ΔE_gap_ value of 3.794 eV that was greater than those of the Cl^-^ and NaCl salt. As can be expected, the Na^+^ ion (*1s*^*2*^*2s*^*2*^*2p*^*6*^), with the electron deficiency, made the graphene more electrophile, while the water molecule made it less electrophile. From Table 4, the electrophilicity indexes (in eV) of **G** and systems **S1–S4** were calculated in the order of S**2** (0.040) < **S1** (0.117) < **G** (0.125) < **S4** (0.175) < **S3** (0.441). Also, the calculated Δε_back-donat._ values revealed that the water molecule made **G** more stable via back donation whereas the Cl^-^ made it less stable. The order of Δε_back-donat._ values (in eV) for systems **S1–S4** was calculated as -0.501 (**S1**) < −0.474 (**S3**) < −0.452 (**S4**) < −0.199 (**S2**). In Table 4, the calculated ionization energy and electron affinity values of H_2_O, Cl-, Na, and NaCl species were given for the prediction/direction of the possible electronic movement between the surface and each of the chemical species. Here the frontier molecular orbital densities for the isolated molecules and designed systems were visualized in Figs. 2 and 3, respectively. As can be seen, the HOMO density for the systems **S2–S4** expanded on the Cl^-^ ion and which was a pictorial proof of the electronic movement from the HOMO of Cl^-^ ions to the LUMO of graphene.

**Table 4:** The chemical reactivity values of the systems **S1**–**S4** and fragments at B3LYP/6-311G(d,p) basis set.

	G	S1	S2	S3	S4	H_2_O	NaCl	Cl^−^	Na^+^
H (−I) (eV)	−5.704	−5.578	−2.108	−8.648	−5.958	−8.148	−6.321	−0.418	−39.519
L (−A) (eV)	−1.686	−1.572	−0.516	−4.854	−2.343	0.780	−2.143	14.606	−6.983
ΔE_gap_ (L–H) (eV)	4.018	4.006	1.592	3.794	3.616	8.928	4.178	15.024	32.536
χ (eV)	−3.695	−3.575	−1.312	−6.751	−4.150	−3.684	−4.232	7.094	−23.251
η (eV)	2.009	2.003	0.796	1.897	1.808	4.464	2.089	7.512	16.268
ω (eV)	0.125	0.117	0.040	0.441	0.175	0.056	0.158	0.123	0.611
ω^+^ (au)	0.066	0.061	0.019	0.326	0.107	0.009	0.089	0.288	0.258
ω− (au)	0.202	0.192	0.068	0.574	0.260	0.144	0.245	0.027	1.113
ΔN_max_ (eV)	1.839	1.785	1.648	3.558	2.296	0.825	2.026	−0.944	1.429
Δε_back_−_donat._ (eV)	−0.502	−0.501	−0.199	−0.474	−0.452	−1.116	−0.522	−1.878	−4.067

To predict the direction of possible electron, transfer between the graphene surface and the considered ions and salts, the energy ranges for each designed system were calculated using the equations below, and the results are given in Table 5. From the results obtained, it can be said that the presence of water adsorbed on the Graphene surface facilitates electron transfer from HOMO to LUMO between the surface and the related chemical species since shortened energy gap. As can be expected from past reports, it is clear from the calculated results that electronic movement from the graphene molecule (HOMO) to the water molecule (LUMO) is more likely. Namely, it was calculated as 6.462 eV and was smaller than that of the inverse scenario (6.484 eV). Without water, the energy gap for the electron movement from the chlorine ion (HOMO) to the graphene surface (LUMO) was determined as 1.154 eV, while it was calculated at 1.268 eV for the same electronic motion in the presence of water. Similarly, the energy gap values for the possible electronic movement from HOMO (Graphene) to LUMO (Na^+^) were calculated as 1.405 eV (without water) > 1.279 eV (with water). On the other hand, for the electrically neutral environment (NaCl salt), it can be said that the electronic movement from the HOMO of Graphene to the LUMO of NaCl is more likely than the opposite version. The energy ranges for these two possible electronic transition scenarios were calculated as follows: ΔE_2_ (3.561 eV) < ΔE_1_ (4.635 eV).

**Table 5:** The energy range values (eV) for the possible electronic movements from HOMO to LUMO for designed systems.

	Without water		With water	
	ΔE_1_	ΔE_2_	ΔE_1_	ΔE_2_
Graphene-H_2_O			6.484	6.462
Graphene-Cl−	1.154	20.184		
Graphene-Na^+^	37.947	1.405		
Graphene-NaCl	4.749	3.435		
(Graphene + H_2_O)-Cl^−^			1.268	20.310
(Graphene + H_2_O)-Na^+^			37.833	1.279
(Graphene + H_2_O)-NaCl			4.635	3.561

*Without water,*

$$\left\{ {\begin{array}{*{20}c} {\left| {\Delta E_{1} = E_{LUMO}^{G} - E_{HOMO}^{{Cl^{ - } }} } \right| \left| {\Delta E_{2} = E_{LUMO}^{{Cl^{ - } }} - E_{HOMO}^{G} } \right|} \\ {\left| {\Delta E_{1} = E_{LUMO}^{G} - E_{HOMO}^{{Na^{ + } }} } \right| \left| {\Delta E_{2} = E_{LUMO}^{{Na^{ + } }} - E_{HOMO}^{G} } \right|} \\ {\left| {\Delta E_{1} = E_{LUMO}^{G} - E_{HOMO}^{NaCl} } \right| \left| {\Delta E_{2} = E_{LUMO}^{NaCl} - E_{HOMO}^{G} } \right|} \\ \end{array} } \right\}$$

*With water*

$$\left\{ {\begin{array}{*{20}c} {\left| {\Delta E_{1} = E_{LUMO}^{G} - E_{HOMO}^{{H_{2} O}} } \right|} & {\left| {\Delta E_{2} = E_{LUMO}^{{H_{2} O}} - E_{HOMO}^{G} } \right|} \\ {\left| {\Delta E_{1} = E_{LUMO}^{S1} - E_{HOMO}^{{Cl^{ - } }} } \right|} & {\left| {\Delta E_{2} = E_{LUMO}^{{Cl^{ - } }} - E_{HOMO}^{S1} } \right|} \\ {\left| {\Delta E_{1} = E_{LUMO}^{S1} - E_{HOMO}^{{Na^{ + } }} } \right|} & \left| {\Delta E_{2} = E_{LUMO}^{{Na^{ + } }} - E_{HOMO}^{S1} } \right| \\ {\left| {\Delta E_{1} = E_{LUMO}^{S1} - E_{HOMO}^{NaCl} } \right|} & \left| {\Delta E_{2} = E_{LUMO}^{NaCl} - E_{HOMO}^{S1} } \right| \\ \end{array} } \right\}$$

## Conclusions

In this study, we attempted to evaluate the presence of water on a solid surface about how higher hydrovoltaic energy can be obtained with which charged ion. Here, the computational results indicate that hydrovoltaic nanogenerators with higher voltage output can be produced on cation-rich water-included surfaces. The adsorption-free energies of systems **S2**–**S4** were determined as **S2** (−52.86) < **S4** (−57.45) < **S3** (−115.86), which indicated the adsorption process could occur spontaneously. Also, the energy range values strongly implied that the charge transfer could be towards from the HOMO of the **S1** to LUMO of the Na^+^ due to the order of it as ΔE_2_ (1.279 eV) < ΔE_1_ (37.833 eV). The results showed that the positively charged Na^+^ ion makes the Graphene-water system have higher energy and thus it is time to be focused on the positively charged G-water systems to produce the next-generation energy.

## Data Availability

All data generated or analyzed during this study are included in this published article.
